# Altered glutamate cysteine ligase activity in peripheral blood mononuclear cells from patients with systemic lupus erythematosus

**DOI:** 10.3892/etm.2014.1689

**Published:** 2014-04-24

**Authors:** WEIQING SONG, JIANGSHUI YUAN, ZONGLIANG ZHANG, LI LI, LIHUA HU

**Affiliations:** 1 Tongji Medical College, Huazhong University of Science and Technology, Wuhan, Hubei 430030, P.R. China; 2 Qingdao Municipal Hospital, Qingdao, Shandong 266071, P.R. China

**Keywords:** glutathione, glutamate cysteine ligase, peripheral blood mononuclear cells, systemic lupus erythematosus, oxidative stress

## Abstract

Reductions in glutathione (GSH) levels have been shown to be associated with aging and the pathogenesis of a variety of diseases, including systemic lupus erythematosus (SLE). Glutamate cysteine ligase (GCL) catalyzes the first and rate-limiting step of GSH synthesis. In order to appraise the correlation between oxidative stress and the severity and activity of SLE, GSH, oxidized GSH (GSSG) and thioredoxin (TRX) concentrations and the enzymatic activity levels of GCL in peripheral blood mononuclear cells (PBMCs) from patients with SLE and healthy controls were studied. In patients with SLE, the levels of GCL activity and GSH decreased, while TRX and GSSG levels increased when compared with those in the healthy controls. GSH concentrations and GCL activity levels negatively correlated with the SLE disease activity index and erythrocyte sedimentation rate. Furthermore, patients with SLE and nephritis had lower levels of GSH and GCL activity and higher levels of TRX and GSSG compared with those in SLE patients without nephritis. Therefore, the results of the present study indicate that insufficient levels of GSH and GCL activity in PBMCs may contribute to the pathogenesis of SLE.

## Introduction

Systemic lupus erythematosus (SLE) is a multifactorial autoimmune disease. The assortment of autoantibodies produced is broad and, as a consequence, the manifestations of the disease are diverse (1). SLE is characterized by arthritis, cutaneous rash, vasculitis, involvement of the central nervous system (CNS) and renal and cardiopulmonary manifestations (2). Reactive oxygen species (ROS) have been considered as risk and enhancer factors for autoimmune diseases (3). Several previous studies have indicated that the chronic immune activation present in the disease is caused by the depletion of intracellular glutathione (GSH) through oxygen-derived free radical production, known as oxidative stress (4–6).

Several clinical conditions, particularly inflammatory and/or immuno-mediated disorders, have been associated not only with oxidative stress in terms of increased ROS formation, but also with impaired antioxidant status mainly in terms of reduced GSH levels and lowered cellular redox potential (7). GSH protects against oxidative stress and detoxifies xenobiotics; thus, is involved in the maintenance of homeostasis. Therefore, reductions in GSH levels are associated with aging and the pathogenesis of a variety of diseases, autoimmune or not, including SLE, rheumatoid arthritis (RA), autoimmune thyroiditis, muscular dystrophy, amyotrophic lateral sclerosis, acquired immunodeficiency syndrome (AIDS), Alzheimer’s, alcoholic liver disease, cataract genesis, respiratory distress syndrome and Werner syndrome (8–11). Altered GSH concentrations may play an important role in a number of autoimmune pathological conditions, prevalently elicited, determined and maintained by inflammatory/immune responses mediated by oxidative stress reactions (12).

In several diseases, including SLE, abnormal T-cell activation and death occurs, which is crucially dependent on the controlled production of ROS and adenosine triphosphate (ATP) in the mitochondria (13). ROS exert their prooxidant activity by inducing tissue damage and the dissociation of iron ions or iron-containing compounds (heme) from a protein-bound state. In addition, the exposure of specific antibodies to heme, transition metal ions or ROS may induce the appearance of new antigen-binding specificities for various autoantigens (14). ROS generation also provides oxidants for thiol oxidation or peroxynitrite formation, which may be a basis for antibody modification (15).

A number of studies have shown that excessive ROS production damages macromolecules, including DNA and proteins, and modulates the expression of a variety of inflammatory molecules, exacerbating inflammation and tissue damage in SLE (16,17). Oxidative damage mediated by ROS results in the generation of deleterious by-products, including aldehydic products, leading to the formation of highly immunogenic adducts with proteins. Thus, pathogenic antibodies are induced in a variety of diseases, including SLE and RA (18).

GSH is synthesized by two consecutive ATP-dependent enzymatic reactions. Glutamate cysteine ligase (GCL) catalyzes the first and rate-limiting step of GSH synthesis. GCL is a heterodimeric enzyme, consisting of a catalytic subunit, (GCLc) and a modulatory subunit (GCLm) that are encoded by two distinct genes (19). GCLc constitutes all the enzymatic activity, but catalytic efficiency is increased substantially by covalent interaction with GCLm (20,21).

Although there is an association between SLE and GSH, GCL activity levels in immune cells from SLE patients remain unclear. Thus, the aim of the present study was to determine the changes in GCL activity in SLE. Thioredoxin (TRX) levels have been shown to increase in response to oxidative stress in experimental (22) and human studies (23). Thus, TRX concentrations were also examined in SLE patients and controls.

The SLE disease activity index (SLEDAI) is a validated model of global assessments from experienced clinicians on disease activity in lupus. It represents the consensus of a group of experts in the field of lupus study (24). Higher SLEDAI scores indicate a greater severity of disease (25). In addition, erythrocyte sedimentation rates (ESR) and autoantibodies are indicators of the degree of inflammation and may be used to monitor disease activity. Therefore, the associations between GSH levels and GCL activity with demographic characteristics, clinical manifestations and laboratory parameters in peripheral blood mononuclear cells (PBMCs) were analyzed in the present study.

## Materials and methods

### Patients and controls

A total of 30 SLE patients of Northern Han Chinese descent, diagnosed according to the criteria of the American College of Rheumatology, were enrolled in the study. Patients were selected from individuals attending outpatient meetings at the Department of Internal Medicine at Qingdao Municipal Hospital (Qingdao, China). In addition, 30 healthy controls were recruited that were ethnicity-, gender- and age-matched with the patients. The patients enrolled were not smokers or alcoholics, and were not associated with any other autoimmune disease. Blood samples from the patients and healthy controls were collected with informed consent from the patients and approval from the Ethics Committee of Qingdao Municipal Hospital. Demographic, laboratory and clinical characteristics of the groups are listed in Table I.

### Preparation of PBMCs

Peripheral blood samples (2 ml), anticoagulated with sodium citrate, were collected from the controls and patients prior to the administration of any immunosuppressive drug. PBMCs were separated by density gradient centrifugation using the Ficoll Paque system (HengXin chemical reagent Co., Ltd., Shanghai, China), according to the manufacturer’s instructions.

### Laboratory measurements

For the patients with SLE, serum levels of C3, C4, IgG, IgA, IgM and C-reactive protein (CRP) were analyzed using an automatic nephelometric immunoassay analyzer (Siemens, Munich, Germany). Autoantibodies, including antinuclear (ANA), anti-dsDNA and anti-Smith (Sm) antibodies, were detected using immunoblotting kits, according to the manufacturer’s instructions (EUROIMMUN AG, Lübeck, Germany).

SLEDAI is a global score reflecting all aspects of disease activity and is a validated model for the assessment of disease activity in SLE (24). Disease activity was determined using the SLEDAI score. ESRs were analyzed using an automatic analyzer (Monitor-J+ analyzer, Vital Diagnostics Srl, Forli, Italy) in all the subjects.

### Measurement of GSH and oxidized glutathione (GSSG) concentration in PBMCs

GSH concentration was measured using a GSH colorimetric assay kit (Beyotime Institute of Biotechnology, Shanghai, China), according to the manufacturer’s instructions. PBMCs were prepared by density gradient centrifugation. The concentration was adjusted to 1×10^7^ cells/ml and the cells were washed in phosphate-buffered saline (PBS). This was followed by centrifugation at 10,000 × g at 4°C for 20 min. Cells in the sediment were collected and mixed with protein removal solution M (v/v, 1/3). The mixtures were vortexed fully and freeze-thawed twice using liquid nitrogen and a water bath. After placing on ice for 5 min, the mixtures were centrifuged at 10,000 × g at 4°C for 10 min. The supernatant was used for GSH detection. For the GSSG assay, GSH was removed with GSH removal liquid, following the manufacturer’s instructions.

### Analysis of TRX levels

PBMCs in PBS were adjusted to a concentration of 1×10^7^ cells/ml and freeze-thawed four times using liquid nitrogen and a water bath. The cells were then centrifuged at 5,000 × g at 4°C for 5 min. The supernatant was used for TRX detection. TRX levels were analyzed using a human TRX ELISA assay kit (Uscn Life Science, Inc., Wuhan, China). Procedures were conducted according to the manufacturer’s instructions.

### GCL activity assay

GCL activity was determined by a fluorescence assay as described by Chen *et al* (26). The concentration of PBMCs was adjusted to 5×10^7^ cells/ml in TES/SB buffer (w/v, 1/4) consisting of 20 mM Tris, 1 mM EDTA, 250 mM sucrose, 20 mM sodium borate and 2 mM serine. The cells were sonicated at 100 W for 60 sec and then centrifuged at 10,000 × g at 4°C for 10 min. The supernatants were collected and centrifuged again at 15,000 × g at 4°C for 20 min. The supernatants were collected and the protein concentrations were determined using a bicinchoninic acid protein assay kit (Beyotime Institute of Biotechnology), with bovine serum albumin used as the standard.

For the GCL activity assay, aliquots of 30 μl supernatant were mixed with 30 μl GCL reaction cocktail (400 mM Tris, 40 mM ATP, 40 mM L-glutamic acid, 2 mM EDTA, 20 mM sodium borate, 2 mM serine and 40 mM MgCl_2_). Following incubation at 37°C for 5 min, 30 μl cysteine solution (30 mM; dissolved in TES/SB buffer) was added and the mixtures were incubated at 37°C for 13 min. The enzymatic reaction in the mixtures was stopped by precipitating proteins with 200 mM 5-sulfosalicylic acid (SSA). After placing on ice for 20 min, the mixtures were centrifuged at 2,000 × g at 4°C for 10 min. Following centrifugation, 20-μl samples of each supernatant containing γ-glutamylcysteine (γ-GC) were added to a 96-well plate designed for fluorescence detection. For each assay, 20 μl γ-GC standards, containing 5 μl GCL reaction cocktail [5 μl SSA (200 mM), 5 μl H_2_O and 5 μl γ-GC standard solution (0, 20, 40, 60, 80, 100, 120 and 140 μM in TES/SB buffer)], was added to each well of the same 96-well plate to generate a standard curve. Next, 180 μl 2,3-naphthalenedicarboxyaldehyde (NDA) was added to each well. Following incubation in the dark at room temperature for 30 min, the formation of NDA-γ-GC was measured (472 nm excitation/528 nm emission) using a fluorescent plate reader (GENios Plus; Tecan, Männedorf Switzerland). The production of γ-GC in each sample was calculated using the standard curve. Values were expressed in mM per min per mg of protein.

### Statistical analysis

Statistical analysis was performed using SPSS software (version 13.0; SPSS, Inc., Chicago, IL, USA). Data are expressed as the mean ± SD. The differences between the subject groups were analyzed using the independent Student’s t-test, while correlation analysis was performed using Spearman’s rank test. P<0.05 was considered to indicate a statistically significant difference. Figures were constructed using GraphPad Prism software (version 5.0; GraphPad Software, Inc., La Jolla, CA, USA).

## Results

### Laboratory measurements of patients with SLE

Demographic characteristics, clinical manifestations and laboratory measurements of the patients with SLE are presented in Table I. Lupus nephritis (LN) is the major indicator of morbidity and mortality in SLE and was identified in 18 of the 30 patients. Arthritis, serositis and CNS disease were identified in 18, 9 and 2 patients, respectively.

A positive result for ANA, anti-dsDNA and anti-Sm autoantibodies was found in 30, 21 and 9 SLE patients, respectively. For the SLE patients, the mean ESR value was 54 mm/h, ranging between 8 and 119 mm/h. In addition, the mean CRP value was 47.2 mg/l with a range between 3 and 101 mg/l. The mean SLEDAI value was 13.16, ranging between 2 and 39.

### Levels of GSH and GSSG in PBMCs from patients with SLE and healthy controls

**Figure 1:**
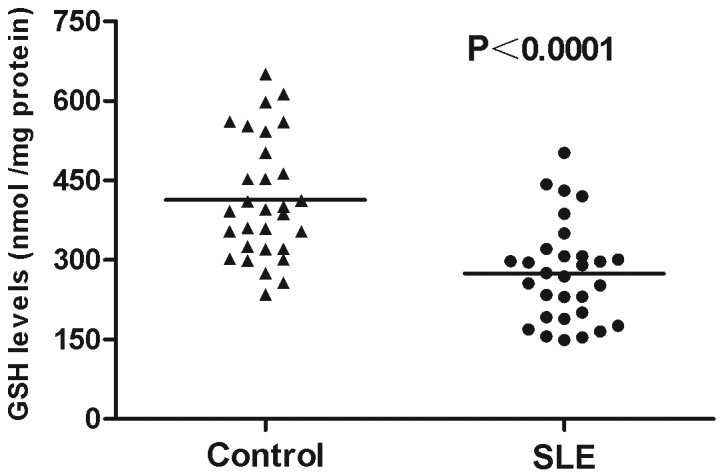
GSH levels in PBMCs from patients with SLE (n=30) and controls (n=30). Horizontal lines indicate mean values (SLE patients, 274.9; controls, 413.6 nmol/mg protein). There was a significant reduction in GSH levels in the SLE patients compared with the controls (P<0.0001). GSH, glutathione; PBMCs, peripheral blood mononuclear cells; SLE, systemic lupus erythematosus.

**Figure 2:**
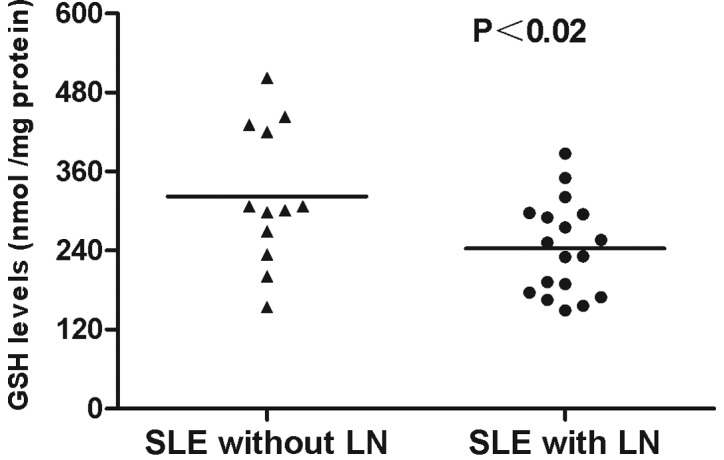
GSH levels in PBMCs from patients with SLE, with (n=18) and without (n=12) LN. Horizontal lines indicate mean values (SLE patients with LN, 243.3; SLE patients without LN, 322.2 nmol/mg protein). There was a significant reduction in GSH levels in the SLE patients with LN compared with those without LN (P<0.02). GSH, glutathione; PBMCs, peripheral blood mononuclear cells; SLE, systemic lupus erythematosus; LN, lupus nephritis.

In order to explore the role of oxidative stress in SLE, GSH and GSSG concentrations and the redox state (GSH/GSSG), were examined in PBMCs from 30 patients with SLE and 30 gender- and age-matched healthy controls. GSH levels considerably decreased in PBMCs from the patients with SLE (274.90±17.08 nmol/mg protein) compared with those in the healthy controls (413.63±20.79 nmol/mg protein; P<0.0001; Fig. 1). By contrast, GSSG levels significantly increased (124.95±4.27 nmol/mg protein; P<0.01) in patients with SLE compared with those in the healthy controls (68.94±1.89 nmol/mg protein; Table II). The reduction in GSH and increase in GSSG concentrations resulted in a decreased redox state (GSH/GSSG) in patients with SLE (2.27±0.43; P<0.001) compared with that in the healthy controls (6.11±1.07; Table II). Furthermore, the levels of the antioxidant GSH were markedly decreased in patients with SLE and LN (243.33±16.73 nmol/mg protein) compared with those in SLE patients without LN (322.25±30.58 nmol/mg protein; P<0.02; Fig. 2). The level of GSSG significantly increased (121.06±8.32 nmol/mg protein; P<0.05) and the redox state markedly decreased (2.01±0.58; P<0.05) in patients with SLE and LN compared with those in SLE patients without LN (Table II).

### TRX levels in PBMCs from patients with SLE and healthy controls

In order to further investigate the status of oxidative stress in SLE, TRX concentrations in the PBMCs from patients with SLE were examined. The average TRX concentration in the patients with SLE was 27.2±9.7 ng/ml, which was significantly increased compared with that in the healthy controls (14.6±7.2; P<0.01; Table II). In addition, the concentration of TRX significantly increased (34.2±5.6; P<0.05) in patients with SLE and LN when compared with the concentration in SLE patients without LN (Table II).

### Changes in the enzymatic activity levels of GCL in PBMCs from patients with SLE and healthy controls

**Figure 3:**
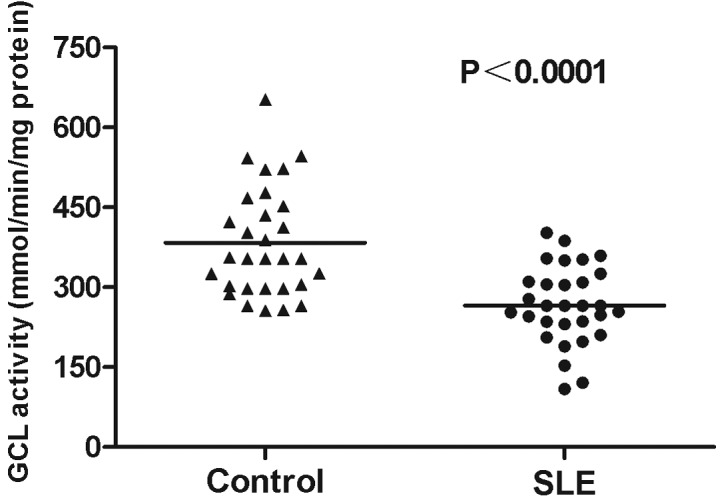
GCL activity levels in PBMCs from patients with SLE (n=30) and controls (n=30). Horizontal lines indicate mean values (SLE patients, 266.1; controls, 383.2 mmol/min/mg protein). There was a significant reduction in GCL activity levels in patients with SLE compared with the controls (P<0.0001). GCL, glutamate cysteine ligase; PBMCs, peripheral blood mononuclear cells; SLE, systemic lupus erythematosus.

**Figure 4:**
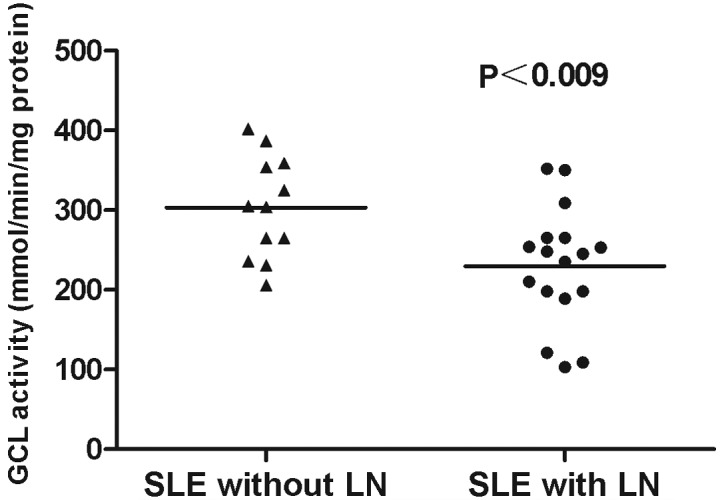
GCL activity levels in PBMCs from patients with SLE, with (n=18) and without (n=12) LN. Horizontal lines indicate mean values (SLE patients with LN, 229.6; SLE patients without LN, 303.2 mmol/min/mg protein). There was a significant reduction in GCL activity levels in SLE patients with LN compared with those without LN (P<0.009). GCL, glutamate cysteine ligase; PBMCs, peripheral blood mononuclear cells; SLE, systemic lupus erythematosus; LN, lupus nephritis.

GCL activity levels in PBMCs from the 30 SLE patients were analyzed. The average GCL activity level in the patients with SLE was 266.10±13.31 mmol/min/mg protein, which was significantly reduced compared with that in the healthy controls (383.27±18.68; P<0.0001; Fig. 3). In addition, GCL activity levels in SLE patients with LN (229.64±17.82) were significantly lower when compared with those in the SLE patients without LN (303.25±18.47; P<0.009; Fig. 4).

### Correlation analysis between GSH levels and characteristics or laboratory parameters in patients with SLE

**Figure 5:**
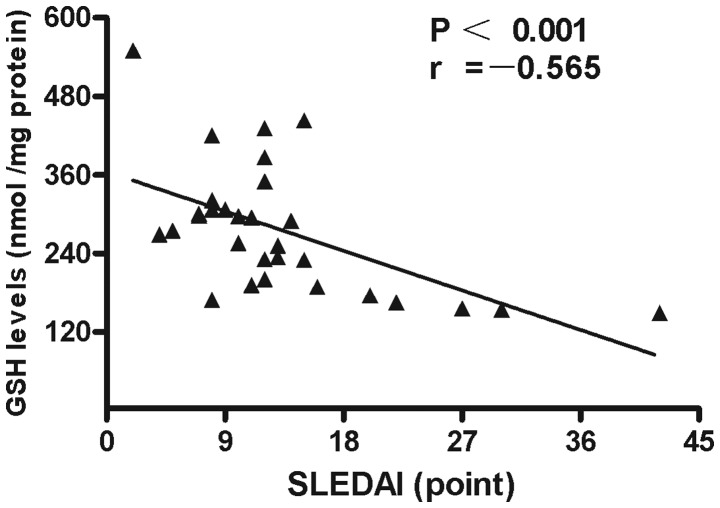
Negative correlation between GSH levels and SLEDAI values in patients with SLE (n=30). SLE, systemic lupus erythematosus; GSH, glutathione; SLEDAI, systemic lupus erythematosus disease activity index.

Associations between GSH levels in PBMCs and demographic characteristics, clinical manifestations and laboratory parameters were analyzed. The results demonstrated that the levels of GSH in the PBMCs negatively correlated with SLEDAI values (r=−0.565; P<0.001; Fig. 5). No statistically significant associations were identified between GSH levels and other characteristics, clinical manifestations or laboratory parameters in the patients with SLE.

### Correlation analysis between GCL activity and characteristics or laboratory parameters in patients with SLE

**Figure 6:**
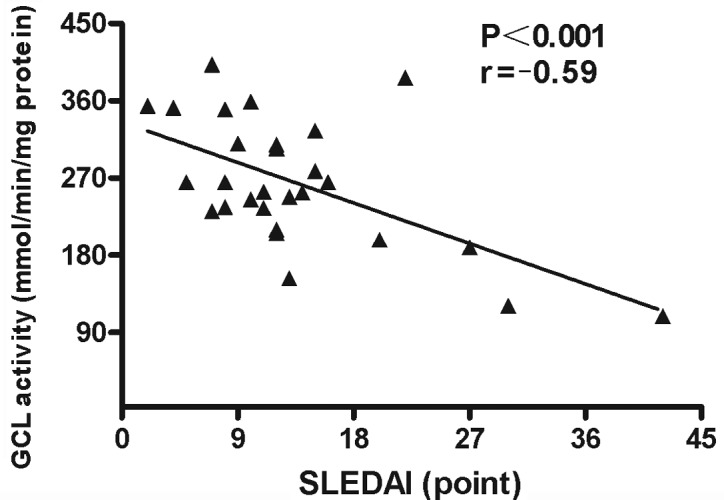
Negative correlation between GCL activity levels and SLEDAI values in patients with SLE (n=30). SLE, systemic lupus erythematosus; GCL, glutamate cysteine ligase; SLEDAI, systemic lupus erythematosus disease activity index.

**Figure 7:**
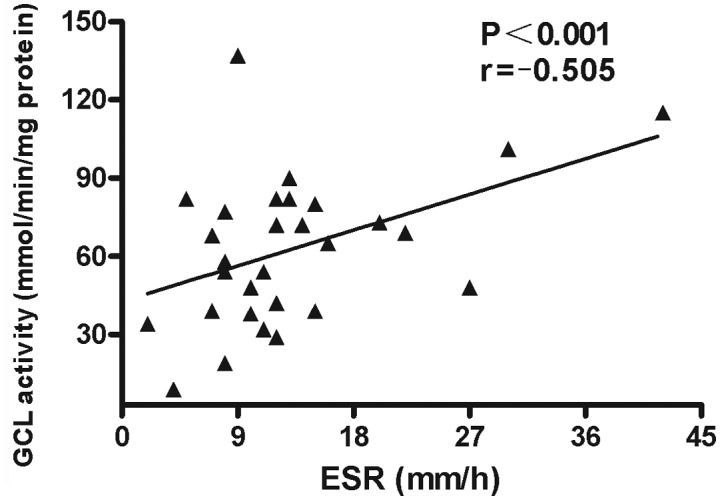
Negative correlation between GCL activity levels and ESRs in patients with SLE (n=30). SLE, systemic lupus erythematosus; GCL, glutamate cysteine ligase; ESR, erythrocyte sedimentation rate.

Associations between GCL activity levels in PBMCs and demographic characteristics, clinical manifestations and laboratory parameters were analyzed. The results revealed that GCL activity levels in PBMCs negatively correlated with SLEDAI scores (r=−0.59; P<0.001; Fig. 6) and ESR (r=−0.505; P<0.001; Fig. 7) in patients with SLE. No statistically significant correlations were identified between GCL activity levels and other characteristics, clinical manifestations or laboratory parameters in the patients with SLE.

## Discussion

Oxidative stress is hypothesized to play a major role in the initiation and progression of autoimmune disease by excessive free radical formation (27). The inability of the antioxidant defense system to cope with oxidative stress is considered to be a possible cause in SLE. The inflammatory nature of the disease indicates that excessive ROS production and an imbalanced redox system may contribute to the immune dysfunction, autoantigen production and perturbation of programmed cell death in SLE (28).

In the present study, GCL activity levels in SLE patients were analyzed, and negative correlations between GCL activity and SLEDAI or ESR were identified. The results clearly indicate that GSH levels and GCL enzymatic activity levels decrease significantly in the PBMCs of patients with SLE, which may be a hallmark of oxidative stress in SLE and coupled together as a cause and consequence in the severity of the disease. The results indicate that GCL activity levels may be involved in disease activity and pathogenesis of SLE.

GCL catalyzes the first and rate-limiting step of GSH synthesis, in which glutamate ligates with cysteine to form γ-GC. This rapidly reacts with glycine to form GSH via the action of GSH synthase (29). The present study demonstrated a good correlation between GSH levels and GCL activity, the primary determinant of the rate of GSH synthesis (30). These observations indicate that a reduction in GCL activity results in a reduction in GSH levels in SLE patients. Consistent with the reduction in GSH levels, GCL activity was inadequate in the PBMCs of patients with SLE. The deficiency of GCL activity and reduction in GSH concentration may contribute to the pathogenesis of SLE through several mechanisms. One possible mechanism is that insufficient GSH content may affect caspase activity, transcription factor activation, Bcl-2 expression and function, thiol-redox signaling and phosphatidylserine externalization, which are early processes in apoptosis (28,31). A negative association of GSH levels with T-lymphocyte and CD4^+^ and CD8^+^ lymphocyte subset apoptosis, and intracellular activated caspase-3 may support the role of GSH in the alteration of apoptosis of T lymphocytes in the SLE disease state (1). These results indicate that GSH is involved in the depletion of CD4^+^ T lymphocytes in patients with SLE (1). The reduction in cellular GSH levels has been attributed mainly to GSH oxidation, promoted by increased production of ROS in the cells (32). It has been reported that GSH depletion in antigen-presenting cells inhibits the production of Th1-related cytokines, including interferon-γ and interleukin-12, and supports the Th2-mediated humoral immune response (33). Since GSH has a significant effect on the ability of the immune system to activate the appropriate Th response, altering the levels of GSH may have significant implications in Th1/Th2-related diseases, including SLE (26). Consequently, insufficient levels of GCL activity and GSH may result in SLE disease, indicating a critical and cell-specific function in the etiology of SLE.

Adequate concentrations of GSH are required for a variety of functions, including the protection of the cell from oxidative damage, the quenching of oxidant species, lymphocyte activation, natural killer cell activation and lymphocyte-mediated cytotoxicity (34,35). A reduction in the level of intracellular GSH correlates with the severity of disease, particularly in patients with LN (36,37).

The inverse correlation between GCL activity, GSH and SLE disease activity and severity, indicated by SLEDAI scores and ESRs, indicates that insufficient levels of GCL activity and GSH may contribute to the severity of disease. Higher SLEDAI scores indicate more severe disease activity (25). Thus, the negative correlation between GCL activity, GSH and SLEDAI scores in patients with SLE indicates that the lower the levels of GCL activity and GSH, the more severe the disease. In addition, ESR is an indicator of the degree of inflammation and is used to monitor disease activity. Since GCL activity was shown to negatively correlate with the ESR in patients with SLE, GCL activity levels may be an index of disease activity. In addition, there was a significant difference in GCL activity and GSH levels between SLE patients with and without LN. Therefore, correlation analysis between GCL activity and SLEDAI, ESR and LN further indicates a potential role of GCL activity and GSH in the pathogenesis of SLE.

TRX is elevated in patients with increased oxidative stress, including AIDS (38) and RA (39). In the present study, increased TRX levels were observed in patients with SLE, which further demonstrates the change in redox state in SLE patients.

In conclusion, GCL enzymatic activity is downregulated and inversely correlates with specific disease parameters in SLE patients. The results indicate that a reduction in GCL activity levels correlates with SLE disease activity and severity. The results support the hypothesis that oxidative stress is a therapeutic target for pharmacological agents in SLE. However, further mechanistic *in vitro* and *in vivo* studies are required to investigate how the interplay between GSH and pathogenesis may lead to the intolerance and aggressiveness of SLE disease activity. Further studies should be directed to evaluate the role of GSH in the pathogenesis of SLE.

## Figures and Tables

**Table I:** Demographic characteristics, clinical features and laboratory measurements of the subjects (n=30 per group).

Characteristics	SLE patients	Healthy controls
Demographic characteristics		
Female, n (%)	24 (80)	23 (76.7)
Male, n (%)	6 (20)	7 (23.3)
Age, years	34.7 (21–57)	35.5 (22–60)
Clinical features		
LN, n (%)	18 (60)	-
Arthritis, n (%)	18 (60)	-
Serositis, n (%)	9 (30)	-
CNS disease, n (%)	2 (6.7)	-
SLEDAI	13.16 (2–39)	-
Laboratory measurements		
C3, g/l	1.10 (0.87–1.7)	
C4, g/l	0.073 (0.07–0.48)	-
IgG, g/l	16.94 (6.8–34.7)	-
IgA, g/l	1.49 (0.68–3.35)	-
IgM, g/l	1.38 (0.32–2.12)	-
ANA, n (%)	30 (100)	-
Anti-dsDNA, n (%)	21 (70)	-
Anti-Sm, n (%)	9 (30)	-
CRP, mg/l	47.2 (3–101)	-
ESR, mm/h	54 (8–119)	9 (4–20)

Except where indicated otherwise, values are the mean (range). SLE, systemic lupus erythematosus; LN, lupus nephritis; CNS, central nervous system; SLEDAI, systemic lupus erythematosus disease activity index; ANA, antinuclear antibody; anti-Sm, anti-Smith antibody; CRP, C-reactive protein; ESR, erythrocyte sedimentation rate.

**Table II:** Oxidant and antioxidant parameters in PBMCs from patients with SLE and healthy controls.

Parameters	Controls	SLE patients	SLE patients with LN	SLE patients without LN
GSH, nmol/mg protein	413.63±20.79	274.90±17.08^c^	243.33±16.73	322.25±30.58^a^
GSSG, nmol/mg protein	68.94±1.89	124.95±4.27^b^	121.06±8.32	147.15±7.51^a^
GSH/GSSG	6.11±1.07	2.27±0.43^c^	2.01±0.58	2.29±0.51^a^
TRX, ng/ml	14.6±7.2	27.2±9.7^b^	34.2±5.6	25.7±6.3^a^

Values are expressed as mean ± SD.

a P<0.05, vs. SLE patients with LN;

b P<0.01 and

c P<0.001, vs. control.

PBMCs, peripheral blood mononuclear cells; SLE, systemic lupus erythematosus; GSH, glutathione; GSSG, oxidized glutathione; TRX, thioredoxin; LN, lupus nephritis.
